# Supplementary material for: Photobiomodulation Therapy in Hypertension Management—Evidence from a Systematic Review and Meta-Analysis
Source: J Clin Med. 2025 Sep 23;14(19):6716. doi: 10.3390/jcm14196716 (PMC12524357; doi:10.3390/jcm14196716)
Supplement: Supplementary file 1 [file jcm-14-06716-s001.zip › Supplementary File S1. Search Strategy.pdf]

Supplementary File S1. Search terms used on PubMed and adapted for other databases

**PubMed Search Terms**
Health condition: (hypertension OR high blood pressure OR arterial hypertension OR hypertensive individual OR hypertensive individuals OR hypertensive patient OR hypertensive patients OR spontaneously hypertensive rats OR two-kidney one-clip hypertension OR two-kidney one clip OR 2K-1C OR hypertensive rat OR hypertensive rats OR essential hypertension OR primary hypertension OR primary hypertensions OR isolated systolic hypertension OR malignant hypertension OR pregnancy-induced hypertension OR pregnancy induced hypertension OR gestational hypertension OR renal hypertension OR renal hypertensions OR renovascular hypertension OR hypertensive crisis OR hypertensive crises OR hypertensive emergency OR hypertensive emergencies OR hypertensive urgency OR hypertensive urgencies OR masked hypertension OR masked hypertensions OR white coat hypertension OR isolated clinic hypertension OR white coat syndrome) AND<br><br>Intervention: (photobiomodulation OR photobiomodulation therapy OR PBM OR PBM TREATMENTS OR PBM therapy OR low-level light therapy OR low-level light therapies OR low level light therapy OR photobiomodulation therapies OR photobiomodulations OR LLLT OR LEDT OR LED Therapy OR low-level laser therapies OR low-power laser irradiation OR low-power laser therapy OR low power laser therapy OR low-power laser therapies OR low power laser therapies OR low-level laser therapy OR low level laser therapy OR low power laser irradiation OR laser biostimulation OR laser phototherapy) AND<br><br>Type of study: (clinical trial OR trial OR randomized trial OR randomised trial OR randomized controlled trial OR randomised controlled trial OR randomized clinical trial OR randomised clinical trial OR rat OR rats OR mice OR mouse OR murinae OR murine OR rattus OR experimental study OR experimental OR animal study OR preclinical study OR preclinical OR experimental design OR animal model OR animal OR animals OR rat model OR rat models)<br>*Filter: Title/Abstract
