# Supplementary material for: Photobiomodulation Therapy in Hypertension Management—Evidence from a Systematic Review and Meta-Analysis
Source: J Clin Med. 2025 Sep 23;14(19):6716. doi: 10.3390/jcm14196716 (PMC12524357; doi:10.3390/jcm14196716)
Supplement: Supplementary file 1 [file jcm-14-06716-s001.zip › Supplementary File S2. Table of excluded articles.pdf]

Supplementary File S2. Rejected papers on abstract analysis (Phase 2)

Author	Title	Reason for rejection
Calò et al. 2004[1]	Increased expression of regulator of G protein signaling-2 (RGS-2) in Bartter's/Gitelman's syndrome. A role in the control of vascular tone and implication for hypertension.	No PBMT
Cassano et al. 2019[2]	Reported Side Effects, Weight and Blood Pressure, After Repeated Sessions of Transcranial Photobiomodulation.	No hypertension
Damush et al. 2013[3]	Implementation of a secondary stroke prevention program: Effect on medication adherence	No PBMT
De Freitas et al. 2022[4]	Effects of light-emitting diode therapy on cardiovascular and salivary nitrite responses in postmenopausal women submitted to a single bout of high-intensity interval training.	No hypertension
De Moraes et al. 2022[5]	Multidrug-resistant protein inhibitor and phosphodiesterase inhibitor potentiate the vasodilator effect induced by photobiomodulation in isolated aortic rings.	Isolated aortic rings
Fierro, Armentano & Silveira 2020[6]	Evaluation of transit time-based models in wearable central aortic blood pressure estimation.	No PBMT
Gonçalves et al. 2020[7]	Evaluation of photobiomodulation in salivar production of patients with anti-hypertensive drug-induced xerostomia.	Conference paper
Hirschberg 2015[8]	Light therapy in treatment of chronic renal disease	Domestic animals
Isabella et al. 2019[9]	Effect of irradiation with intravascular laser on the hemodynamic variables of hypertensive patients: Study protocol for prospective blinded randomized clinical trial.	Only study protocol
Semplicini et al. 2006[10]	Reduced expression of regulator of G-protein signaling 2 (RGS2) in hypertensive patients increases calcium mobilization and ERK1/2 phosphorylation induced by angiotensin II.	No PBMT
Syed et al. 2023[11]	Photobiomodulation therapy mitigates cardiovascular aging and improves survival.	No hypertension
Valverde et al. 2022[12]	Photobiomodulation for Hypertension and Alzheimer's Disease.	Review study

Wassmann et al. 2001[13]	Endothelial dysfunction and oxidative stress during estrogen deficiency in spontaneously hypertensive rats.	No PBMT
Yang et al. 2018[14]	Correlation analysis between peripheral blood monocyte subsets and left ventricular remodeling in spontaneously hypertensive rats.	No PBMT

Rejected papers on full-text analysis (Phase 3)

Author	Title	Reason for rejection
Buzinari et al. 2022[15]	Nitric oxide storage levels modulate vasodilation and the hypotensive effect induced by photobiomodulation using an aluminum gallium arsenide (AlGaAs) diode laser (660 nm).	Used NOS inhibitor to prove the hypotensive effect depends on NO, but there is no control group without the use of the inhibitor
Canal et al. 2013[16]	Hemodynamic changes post low level laser therapy in elderly obese rats: An experimental study	Conference paper
De Moraes et al. 2020[17]	Energy-dependent effect trial of photobiomodulation on blood pressure in hypertensive rats.	No control group
Elmahy, Mohamed & Rashed 2016[18]	Influence of laser puncture on endothelial dysfunction on hypertensive patients	No isolated PBMT. Only groups with PBMT plus exercise and exercise associated to sham PBMT
Madi et al. 2016[19]	The immediate hemodynamic response post laser therapy in hypertensive and normotensive pregnant woman	Conference paper
Oishi et al. 2017[20]	Hypotensive acute effect of photobiomodulation therapy on hypertensive rats	No control group
Silva et al. 2015[21]	LOW LEVEL LASER THERAPY IMPROVES CARDIOVASCULAR AUTONOMIC ACTIVITY IN SPONTANEOUSLY HYPERTENSIVE RATS	Conference paper
Kovalenko et al. 2019[22]	Correction of functional capacity of myocardium in arterial hypertension associated with hyperuricemia and poikilocytosis comorbidities	Full-text not found

Achilov et al. 2010[23]	Potentials of combined non-medication therapy of arterial hypertension associated with ischemic heart disease	Full-text not found
Kucheriavyĭ, Ponomarenko & Kovlen, 2007[24]	Magnetolaser therapy of patients with bronchial asthma in combination with essential hypertension	Full-text not found
Antoniuk et al. 2011[25]	Combined application of magnetolaserotherapy and polyunsaturated fatty acids for the treatment of patients with hypertensive disease	Full-text not found
Ucero et al. 2013[26]	Laser Therapy in Metabolic Syndrome-Related Kidney Injury	Metabolic Syndrome model
Pandey, Lytle & Mishra, 2013[27]	Low Level Laser Therapy (Lllt) For the Treatment of Hypertension	Insufficient data about inclusion criteria (number of participans per group, age, frequency of treatment, protocol..)

References:

1. Calò, L. A., Pagnin, E., Davis, P. A., Sartori, M., Ceolotto, G., Pessina, A. C., & Semplicini, A. Increased expression of regulator of G protein signaling-2 (RGS-2) in Bartter's/Gitelman's syndrome. A role in the control of vascular tone and implication for hypertension. The Journal of clinical endocrinology and metabolism. **2004**, 89(8), 4153–4157. https://doi.org/10.1210/jc.2004-0498.
2. Cassano, P., Caldieraro, M. A., Norton, R., Mischoulon, D., Trinh, N. H., Nyer, M., Dording, C., Hamblin, M. R., Campbell, B., & Iosifescu, D. V. Reported Side Effects, Weight and Blood Pressure, After Repeated Sessions of Transcranial Photobiomodulation. Photobiomodulation, photomedicine, and laser surgery. **2019**, 37(10), 651–656. https://doi.org/10.1089/photob.2019.4678.
3. Damush, T., Myers, L., Anderson, J., Yu, Z., Ofner, S., Schmid, A., & Williams, L. Abstract TP415: Implementation of a Secondary Stroke Prevention Program: Effect on Medication Adherence. Stroke. **2013**, 44(suppl_1), ATP415-ATP415. https://doi.org/doi:10.1161/str.44.suppl_1.ATP415.
4. de Freitas, V. H., Mariano, I. M., Amaral, A. L., Rodrigues, M. L., Carrijo, V. H. V., & Puga, G. M. Effects of light-emitting diode therapy on cardiovascular and salivary nitrite responses in postmenopausal women submitted to a single bout of high-intensity interval training. *Lasers in medical Science.* **2022**, *37*(6), 2655–2665. https://doi.org/10.1007/s10103-022-03533-z.
5. de Moraes, L. H. O., Terroni, B., da Silva Mayer, N. F., & Rodrigues, G. J. Multidrug-resistant protein inhibitor and phosphodiesterase inhibitor potentiate the vasodilator effect induced by photobiomodulation in isolated aortic rings. *Lasers in medical Science.* **2022**, *37*(2), 1209–1216. https://doi.org/10.1007/s10103-021-03374-2.

6. Fierro, G., Armentano, R., & Silveira, F. Evaluation of transit time-based models in wearable central aortic blood pressure estimation. *Biomedical physics & engineering express*. **2020**, *6*(3), 035006. https://doi.org/10.1088/2057-1976/ab7a55.
7. Goncalves, M.L.Z.V.M.L.L, Da Silva, C. D., Barbosa Filho, V.F., Lourenco, M. F., Bussadori, S. K., Deana, A. M. Evaluation of photobiomodulation in salivar production of patients with anti-hypertensive drug-induced xerostomia. *Lasers in medical science*. **2020**; 35(1): 280.
8. Hirschberg R.E. Light therapy in treatment of chronic renal disease, 28th International Congress Laser Medicine and IALMS Courses, Laser Florence 2014. *Lasers in Medical Science*. **2015**, http://dx.doi.org/10.1007/s10103-015-1805-7.
9. Isabella, A. P. J., Silva, J. T. C., da Silva, T., Rodrigues, M. F. S. D., Horliana, A. C. R. T., Motta, L. J., Bussadori, S. K., Pavani, C., & Silva, D. F. T. D. Effect of irradiation with intravascular laser on the hemodynamic variables of hypertensive patients: Study protocol for prospective blinded randomized clinical trial. *Medicine*. **2019**, 98(14), e15111. https://doi.org/10.1097/MD.0000000000015111.
10. Semplicini, A., Lenzini, L., Sartori, M., Papparella, I., Calò, L. A., Pagnin, E., Strapazzon, G., Benna, C., Costa, R., Avogaro, A., Ceolotto, G., & Pessina, A. C. Reduced expression of regulator of G-protein signaling 2 (RGS2) in hypertensive patients increases calcium mobilization and ERK1/2 phosphorylation induced by angiotensin II. *Journal of hypertension*. **2006**, 24(6), 1115–1124. https://doi.org/10.1097/01.hjh.0000226202.80689.8f.
11. Syed, S. B., Ahmet, I., Chakir, K., Morrell, C. H., Arany, P. R., & Lakatta, E. G. Photobiomodulation therapy mitigates cardiovascular aging and improves survival. *Lasers in surgery and medicine*. **2023**, 55(3), 278–293. https://doi.org/10.1002/lsm.23644.
12. Valverde, A., & Mitrofanis, J. Photobiomodulation for Hypertension and Alzheimer's Disease. Journal of Alzheimer's disease : *JAD*. **2022**, 90(3), 1045–1055. https://doi.org/10.3233/JAD-220632.
13. Wassmann, S., Bäumer, A. T., Strehlow, K., van Eickels , M., Grohé, C., Ahlbory, K., Rösen, R., Böhm, M., & Nickenig, G. Endothelial dysfunction and oxidative stress during estrogen deficiency in spontaneously hypertensive rats. *Circulation*. **2001**, 103(3), 435–441. https://doi.org/10.1161/01.cir.103.3.435.
14. Yang, G.; Zhou, X.; Liu, J.; Sun, J.; Sun, H.; Zhao, J.; Jiang, T.; Li, Y.. A4894 Correlation Analysis Between Peripheral Blood Monocyte Subsets and Left Ventricular Remodeling in Spontaneously Hypertensive Rats. *Journal of Hypertension*. **2018**, 36():p e38. DOI: 10.1097/01.hjh.0000548140.86629.48.
15. Buzinari, T. C., de Moraes, T. F., Conceição-Filho, J. C., Cárnio, E. C., Almeida-Lopes, L., Salgado, H. C., & Rodrigues, G. J. Nitric oxide storage levels modulate vasodilation and the hypotensive effect induced by photobiomodulation using an aluminum gallium arsenide (AlGaAs) diode laser (660 nm). *Lasers in medical science*. **2022**, 37(6), 2753–2762. https://doi.org/10.1007/s10103-022-03551-x.

16. Canal M., Conti F., Pinto N., Pinto M., Silva B., Sanches I., Duarte I., De Angelis K. Hemodynamic changes post low level laser therapy in elderly obese rats: An experimental study, 33rd Annual Conference of the American Society for Laser Medicine and Surgery, ASLMS 2013. *Lasers in Surgery and Medicine*. **2013**. http://dx.doi.org/10.1002/lsm.22127.
17. De Moraes, T. F., Filho, J. C. C., Oishi, J. C., Almeida-Lopes, L., Parizotto, N. A., & Rodrigues, G. J. Energy-dependent effect trial of photobiomodulation on blood pressure in hypertensive rats. *Lasers in medical science*. **2020**, 35(5), 1041–1046. https://doi.org/10.1007/s10103-019-02883-5.
18. Elmahy, R. M. A., Mohamed, G. S. & Rashed, L. A. Influence of Laser Puncture on Endothelial Dysfunction on Hypertensive Patients. *Trends in Medical Research*. **2016**, 11: 107-112.
19. Madi O., Tomimura S., Pinto N., Duarte I., Lopes H., Colombo F., Chavantes M.C. The immediate hemodynamic response post laser therapy in hypertensive and normotensive pregnant woman, 36th Annual Conference of the American Society for Laser Medicine and Surgery, ASLMS 2016. *Lasers in Surgery and Medicine.* **2016**. http://dx.doi.org/10.1002/lsm.22527.
20. Oishi, J. C., De Moraes, T. F., Buzinari, T. C., Cárnio, E. C., Parizotto, N. A., & Rodrigues, G. J. Hypotensive acute effect of photobiomodulation therapy on hypertensive rats. *Life sciences*. **2017**, *178*, 56–60. https://doi.org/10.1016/j.lfs.2017.04.011.
21. Silva, B., Tomimura, S., Sanches, I., Canal, M., Pinto, N., Madi, O., Conti, F., Angelis, K., Colomb, F., Chavantes, M. C. Low Level Laser Therapy Improves Cardiovascular Autonomic Activity in Spontaneously Hypertensive Rats. *Lasers in Surgery and Medicine*. **2015**, 47 (4), 382-383.
22. Kovalenko, Y. L., Melekhovets, O. K., Orlovskyi, V. F., & Melekhovets, Y. V. Correction of functional capacity of myocardium in arterial hypertension associated with hyperuricemia and poikilocytosis comorbidities. *Zaporozhye Medical Journal*. **2019**, *21*(4). https://doi.org/10.14739/2310-1210.2019.4.173151.
23. Achilov, A. A., Lebedeva, O. D., Bulatetskaia, L. S., Usmonzoda, D. U., Belov, A. S., Kotov, S. A., Achilova, S.hA., & Rykov, S. V. *Voprosy kurortologii, fizioterapii, i lechebnoi fizicheskoi kultury*. **2010**, (6), 12–15.
24. Kucheriavyĭ, A. M., Ponomarenko, G. N., & Kovlen, D. V. *Voprosy kurortologii, fizioterapii, i lechebnoi fizicheskoi kultury*. **2007**, (2), 4–7.
25. Antoniuk, M. V., Kantur, T. A., Karaman, I.uK., & Zhukova, N. V. *Voprosy kurortologii, fizioterapii, i lechebnoi fizicheskoi kultury*. **2011**, (1), 9–13.
26. Ucero, A. C., Sabban, B., Benito-Martin, A., Carrasco, S., Joeken, S., & Ortiz, A. Laser therapy in metabolic syndrome-related kidney injury. *Photochemistry and photobiology*. **2013**, *89*(4), 953–960. https://doi.org/10.1111/php.12055.
27. Pandey, R., Lutle, L., Mishra, A. Low Level Laser Therapy (Lllt) For the Treatment of Hypertension. *International Journal of Engineering Research and Development*. **2013**, 6. 35-38.
