# Supplementary material for: Photobiomodulation Therapy in Hypertension Management—Evidence from a Systematic Review and Meta-Analysis
Source: J Clin Med. 2025 Sep 23;14(19):6716. doi: 10.3390/jcm14196716 (PMC12524357; doi:10.3390/jcm14196716)
Supplement: Supplementary file 1 [file jcm-14-06716-s001.zip › Supplementary File S3. PEDro Scale.pdf]

Supplementary File S3. Assessment of risk of bias of included articles (PEDro Scale)

Study	Random Allocation	Concealed Allocation	Similarity at Baseline	Blinding of subjects	Blinding of therapists	Blinding of assessors	Sample loss	Intention to treat	Between-group comparisons	Measures of variability	Total
Awad, Ibrahim & Gabr (2013)	1	0	1	0	0	0	0	1	1	1	**5**
Hamed & Maghraby (2010)	1	0	1	0	0	0	0	0	1	1	**4**
Pereira et al (2018)	1	1	1	1	1	1	0	0	0	1	**7**
Zhang et al (2008)	1	0	1	1	0	0	1	0	0	1	**5**

References: Awad, M.A.; Ibrahim, D.A.; Gabr, A.A. Effect of Laser Acupuncture on Reducing Postmenopausal Hypertension. Bull. Fac. Phys. Ther. 2013, 18, 109–117.; Hamed, H.M.; Maghraby, M.A.A. Effectiveness of Laser Acupoint Therapy and Exercise Program on Oxidative Stress and Antioxidant Response in Mild Essential Hypertensive Patients. Bull. Egypt. Soc. Physiol. Sci. 2010, 30, 229–244.; Pereira, R.D.M.; Alvim, N.A.T.; Pereira, C.D.; Gomes Junior, S. Laser acupuncture protocol for essential systemic arterial hypertension: Randomized clinical trial. Rev. Lat. Am. Enferm. 2018, 26, e2936. https://doi.org/10.1590/1518-8345.1887.2936.; Zhang, J.; Marquina, N.; Oxinos, G.; Sau, A.; Ng, D. Effect of laser acupoint treatment on blood pressure and body weight-a pilot study. J. Chiropr. Med. 2008, 7, 134–139.
