# Supplementary material for: Photobiomodulation Therapy in Hypertension Management—Evidence from a Systematic Review and Meta-Analysis
Source: J Clin Med. 2025 Sep 23;14(19):6716. doi: 10.3390/jcm14196716 (PMC12524357; doi:10.3390/jcm14196716)
Supplement: Supplementary file 1 [file jcm-14-06716-s001.zip › Supplementary File S4. GRADE assessment.pdf]

Supplementary File S4. Certainty of the evidence of randomized controlled trials

Question: PBMT + antihypertensive drugs compared to sham PBMT + antihypertensive drugs for hypertension

Certainty assessment							№ of patients		Effect		Certainty	Importance
№ of studies	Study design	Risk of bias	Inconsistency	Indirectness	Imprecision	Other considerations	PBMT + antihypertensive drugs	Sham PBMT + antihypertensive drugs	Relative (95% CI)	Absolute (95% CI)		
**SBP**												
1	randomized trials	not serious	not serious	not serious	extremely serious[a]	none	43	36	-	-	⊕◯◯◯ Very low[a]	CRITICAL
**DBP**												
1	randomized trials	not serious	not serious	not serious	extremely serious[a]	none	43	36	-	-	⊕◯◯◯ Very low[a]	CRITICAL

Question: PBMT + Captopril-25 mg compared to Captopril-25 mg for hypertension

Certainty assessment							№ of patients		Effect		Certainty	Importance
№ of studies	Study design	Risk of bias	Inconsistency	Indirectness	Imprecision	Other considerations	PBMT + Captopril-25 mg	Captopril-25 mg	Relative (95% CI)	Absolute (95% CI)		
**SBP**												
1	randomized trials	serious[b]	not serious	not serious	extremely serious[a]	none	30	30	-	-	⊕◯◯◯ Very low[a,b]	CRITICAL
**DBP**												
1	randomized trials	serious[b]	not serious	not serious	extremely serious[a]	none	30	30	-	-	⊕◯◯◯ Very low[a,b]	CRITICAL

Question: PBMT compared to Control for hypertension

Certainty assessment							№ of patients		Effect		Certainty	Importance
№ of studies	Study design	Risk of bias	Inconsistency	Indirectness	Imprecision	Other considerations	PBMT	Control	Relative (95% CI)	Absolute (95% CI)		
**SBP**												
1	randomized trials	serious[c]	not serious	not serious	extremely serious[a]	none	15	15	-	-	⊕◯◯◯ Very low[a,c]	CRITICAL
**DBP**												

Certainty assessment							№ of patients		Effect		Certainty	Importance
№ of studies	Study design	Risk of bias	Inconsistency	Indirectness	Imprecision	Other considerations	PBMT	Control	Relative (95% CI)	Absolute (95% CI)		
1	randomized trials	serious[c]	not serious	not serious	extremely serious[a]	none	15	15	-	-	⊕◯◯◯ Very low[a,c]	CRITICAL
**HR**												
1	randomized trials	serious[c]	not serious	not serious	extremely serious[a]	none	15	15	-	-	⊕◯◯◯ Very low[a,c]	CRITICAL

Question: PBMT compared to Sham PBMT for hypertension

Certainty assessment							№ of patients		Effect		Certainty	Importance
№ of studies	Study design	Risk of bias	Inconsistency	Indirectness	Imprecision	Other considerations	PBMT	Sham PBMT	Relative (95% CI)	Absolute (95% CI)		
**SBP**												
1	randomized trials	serious[d]	not serious	not serious	extremely serious[a]	none	23	22	-	-	⊕◯◯◯ Very low[a,d]	CRITICAL
**DBP**												
1	randomized trials	serious[d]	not serious	not serious	extremely serious[a]	none	23	22	-	-	⊕◯◯◯ Very low[a,d]	CRITICAL

CI: Confidence Interval
SBP: Systolic Blood Pressure
DBP: Diastolic Blood Pressure
HR: Heart Rate

Explanations

a. The evidence contains only one study, being lower than the Optimal Information Size (OIS);
b. Awad, Ibrahim & Gabr (2013) study had a lack of concealed allocation and blinding of both subjects, therapists and assessors;
c. Hamed & Maghraby (2010) study had a grade 4 at PEDro Scale. The study had a lack of concealed allocation, blinding of subjects, therapists and assessors;
d. Zhang et al (2008) study had a grade 5 at PEDro Scale. The study had a lack of concealed allocation, blinding of therapists and assessors and between-group comparisons.

Certainty of the evidence of experimental studies

Question: PBMT compared to Sham PBMT for hypertension

Certainty assessment							№ of patients		Effect		Certainty	Importance
№ of studies	Study design	Risk of bias	Inconsistency	Indirectness	Imprecision	Other considerations	PBMT	Sham PBMT	Relative (95% CI)	Absolute (95% CI)		
**SBP**												

Certainty assessment							№ of patients		Effect		Certainty	Importance
№ of studies	Study design	Risk of bias	Inconsistency	Indirectness	Imprecision	Other considerations	PBMT	Sham PBMT	Relative (95% CI)	Absolute (95% CI)		
4	experimental study	serious[a]	not serious	very serious[b]	serious[c]	none	65	50	-	see comment	⊕◯◯◯ Very low[a,b,c]	CRITICAL
**DBP**												
2	experimental study	serious[d]	not serious	very serious[b]	serious[c]	none	34	34	-	see comment	⊕◯◯◯ Very low[b,c,d]	CRITICAL
**MAP**												
2	experimental study	serious[d]	not serious	very serious[b]	serious[c]	none	34	34	-	see comment	⊕◯◯◯ Very low[b,c,d]	CRITICAL
**HR**												
2	experimental study	serious[d]	not serious	very serious[b]	serious[c]	none	34	34	-	see comment	⊕◯◯◯ Very low[b,c,d]	IMPORTANT
**NO**												
3	experimental study	serious[d]	not serious	very serious[b]	serious[c]	none	57	42	-	see comment	⊕◯◯◯ Very low[b,c,d]	IMPORTANT

Question: PBMT compared to Control for hypertension

Certainty assessment							№ of patients		Effect		Certainty	Importance
№ of studies	Study design	Risk of bias	Inconsistency	Indirectness	Imprecision	Other considerations	PBMT	Control	Relative (95% CI)	Absolute (95% CI)		
**SBP**												
1	experimental study	not serious	not serious	very serious[b]	serious[c]	none	8	8	-	-	⊕◯◯◯ Very low[b,c]	CRITICAL
**NO**												
1	experimental study	not serious	not serious	very serious[b]	serious[c]	none	8	8	-	-	⊕◯◯◯ Very low[b,c]	IMPORTANT

CI: Confidence Interval
SBP: Systolic Blood Pressure
DBP: Diastolic Blood Pressure
MAP: Mean Arterial Pressure
HR: Heart Rate
NO: Nitric Oxide

Explanations

a. One of the studies was rated as having high risk of bias, and the two other had a classification of unclear risk of bias;
b. There is very serious indirectness due to the study designs being experimental studies;
c. The number of animals is lower than the Optimal Information Size;
d. One of the studies was rated as having high risk of bias, and the other one had a classification of unclear risk of bias.
